# A tissue-specific gene expression template portrays heart development and pathology

**DOI:** 10.1186/1479-7364-8-6

**Published:** 2014-03-11

**Authors:** Amy Rodemoyer, Nataliya Kibiryeva, Alexis Bair, Jennifer Marshall, James E O’Brien, Douglas C Bittel

**Affiliations:** 1 Kansas City University of Medicine and Biosciences, Kansas, MO 64106, USA; 2 The Ward Family Heart Center, Children's Mercy Hospitals and Clinics, Kansas City, MO 64108, USA; 3 University of Missouri-Kansas City School of Medicine, 5100 Rockhill Rd, Kansas City, MO 64110, USA

**Keywords:** Gene expression template, Congenital heart defect, Tetralogy of Fallot

## Abstract

Congenital heart defects (CHD) are the most common cause of death in children under the age of 1. Tetralogy of Fallot (TOF) is a severe CHD that results from developmental defects in the conotruncal outflow tract. Recently, a tissue-specific gene expression template (GET) was derived from microarray data that accurately characterized multiple normal human tissues. We used the GET to examine spatial, temporal, and a pathological condition (TOF) within a single organ, the heart. The GET, as previously defined, generally identified temporal and spatial differences in the cardiac tissue. Differences in the stoichiometry of the GET reflected the severe developmental disturbance associated with TOF. Our analysis suggests that the homoeostatic equilibrium assessed by the GET at the inter-organ level is generally maintained at the intra-organ level as well.

## Background

With the advent of tools for whole transcriptome analysis, there has been great interest in defining the gene expression profile of normal tissues as well as pathological states in order to understand the genetic regulation of development and identify potential therapeutic targets [1-5]. These studies have produced insight into the complexity of temporal and spatial regulation of gene expression. They also illustrate the interconnected nature of genes, forming pathways that are themselves interconnected.

Congenital heart defects (CHD) are the most common birth defect and are the highest cause of mortality in children under the age of 1. Tetralogy of Fallot (TOF) is a severe constellation of defects that results from a disruption of the communication pathways between the first and second heart fields at about 20 days of gestation. A multitude of genes and genetic networks contribute to the spatial and temporal specification required for proper embryological heart formation [6-11]. Variation in the expression of cardiac genes or genes involved in network buffering, variation in the expression of microRNAs, and/or methylation differences or differences in gene transcript processing, could all cause shifts in pathway function leading to failure of the tight control of cell lineage specification required for normal heart development. However, even with the sophisticated tools that allow whole genome analysis, we still know relatively little about the genetic causes of TOF, as is the case for most CHDs.

The complexity of the cardiac regulatory system controlling heart development is challenging, but it was recently shown that a gene expression template (GET) containing only 56 genes could be used as a molecular signature to accurately specify at least 24 different disease-free tissues [12], including the heart. Hwang et al. [12] suggested that tissues preserve normal functioning and morphology by maintaining specific stoichiometry among genes and genetic networks. The state of the biochemical tissue-specific stoichiometry can be portrayed by the expression pattern of a compact set of representative genes that may reflect the status of key regulatory pathways. The pattern of expression of the 56 genes in the GET could uniquely identify 24 distinct tissue types. Interestingly, they looked at the developmental behavior of the GET by analyzing expression in cultured cells and two embryonic tissues, the lung and skin. These analyses suggested that embryonic tissues have a distinct GET stoichiometry that progresses to the terminal equilibrium as the developmental stage progresses. Moreover, it would be expected that the significant deviation from normal stoichiometry, as assessed by the GET, would result in malfunction or abnormal growth of cells and tissues. They demonstrated that their GET model could be used to differentiate a pathologic state (cancer) from the normal tissue. Therefore, they concluded that the change in the expression pattern of the 56 genes that comprise their GET reflected the deviation in biochemical stoichiometry resulting in abnormal growth.

We wondered if the expression pattern of the 56 genes comprising the GET defined by Hwang et al. [12] would produce expression patterns that were unique to the different regions of the heart, or to different time points in development of the heart, or to a pathological state (TOF). Therefore, using this set of 56 genes, we examined temporal and spatial changes in expression in normally developing heart tissue and the expression pattern in the right ventricular tissue from children with TOF. The significant variation seen in the pathologic tissue demonstrates that the GET can be used to characterize tissue-specific pathologic states as well as temporal and spatial differences in normally developing cardiac tissue.

## Results and discussion

Hwang et al. [12] used Affymetrix gene expression arrays (Affymetrix Inc., Santa Clara, CA, USA) to derive tissue-specific expression patterns that could distinguish at least 24 distinct tissues using as few as 56 genes, creating a GET. We previously used whole genome expression microarrays to analyze right ventricular tissue from infants with TOF, normally developing-age-matched infants and three fetuses at approximately 90 days of gestation [13]. In addition, the Gene Expression Omnibus contained gene expression microarray data for adult left and right ventricles and the sigmoid colon, which we downloaded (see Additional file 1: Table S1 for a complete listing of the original array data, as well as the source). We extracted the expression levels for these 56 genes from our cardiac expression data [13] and the cardiac array data downloaded from the Gene Expression Omnibus (GEO) to assess the GET in different regions of the heart, at different time points during heart development, and in a pathological condition, tetralogy of Fallot (TOF).

**Figure 1:**
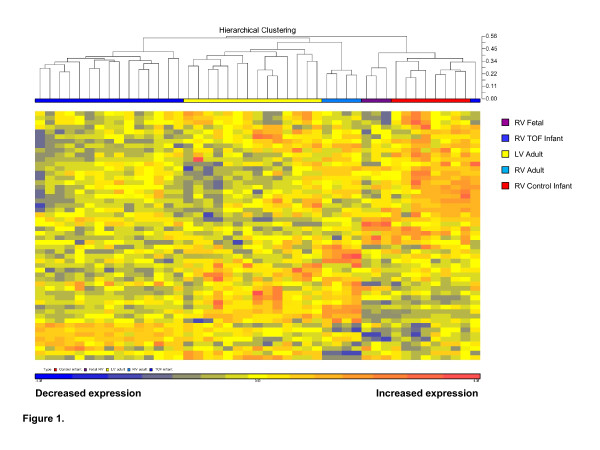
**Hierarchical clustering based on 54 genes of the gene expression template (GET).** The clustering algorithm used the complete linkage algorithm (i.e., point-to-point distance of furthest neighbors). Changes in expression are based on comparisons to the average values from samples from the normally developing infants.

Of the 56 genes used by Hwang et al., we were able to identify 54 containing core probes on the exon arrays (genes are listed in Additional file 2: Table S2). The two genes that did not have core probes on our arrays were HLC-A and MOBP, and we chose not to include them in the clustering analysis as they were inadequately sampled on any of the arrays. We used the remaining 54 genes to generate an unsupervised hierarchical dendrogram of the data derived from the heart tissue (Figure 1, clustering was based on a complete linkage algorithm, see Section ‘Methods’ for explanation). Next, we looked at the statistically significant changes in gene expression (*t* test with Benjamini-Hochburg correction for multiple testing) in pairwise assessments among groups in order to determine what genes and/or pathways were driving divergence among sets of tissues (Additional file 2: Table S2). Three genes, PRKCB, TFPI2, and CA2, did not have significant changes in expression in any of the comparisons we made. Regardless, the dendrogram was generated using all 54 genes from the GET derived by Hwang et al. [12].

As seen in Figure 1, the GET produced a dendrogram in which individuals were assembled into groups that reflected the temporal and spatial characteristics of each group. The temporal stage (mature vs. young) was the most important component in clustering the samples as mature right ventricle (RV) and left ventricle (LV) clustered more closely together compared to fetal or infant LV. The TOF samples also clustered independently indicating that pathology was an important component in generating the dendrogram. We also included the expression pattern of genes from the colon as an outgroup comparison due to its different embryological origin from that of cardiac tissue (Additional file 3: Figure S1). As expected, there was a distinct difference in clustering between the heart tissue and the colon tissue. Organ type was a much greater clustering component than either spatial or pathological differences in expression of the GET.

Each tissue from within the heart clustered according to spatial, temporal, or pathological conditions, supporting the theory of tissue/cell type specificity previously set forth by Hwang et al. We had a single exception to this, which was one sample from an infant with TOF that clustered with the group of normal infant RV. The medical record of this infant did not indicate any unusual differences compared to the other infants with TOF which might have explained the deviation in expression pattern. The GET pattern in this infant likely reflects less perturbation in the GET than that seen in the other infants with TOF.

**Figure 2:**
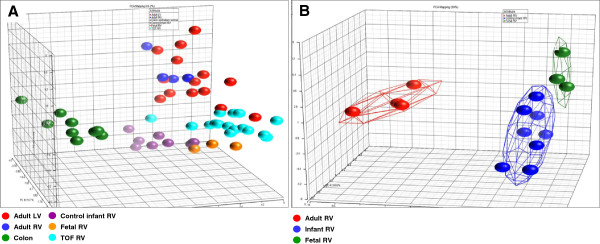
**Principal component analysis of the expression pattern of the GET within. (A)** All groups examined. **(B)** Fetal, infant, and adult right ventricular tissues (all normally developing with no known defects). The *colored connected lines* form a cloud around the group at two standard deviations.

In addition, principal component analysis (PCA) clearly demonstrated that the variability between the tissues (colon vs. heart) was greater than the intra-tissue variability (Figure 2A). The PCA produced a clear grouping of cardiac tissue according to common origin. In the adult tissues examined, adult RV and adult LV were more closely related to each other than to infant or fetal tissues, again indicating that age (mature vs. fetal or infant tissue) influenced expression of the GET to a greater extent than did spatial origin. PCA placed the samples from infants with TOF clustered graphically between the adult and infant tissues.

We analyzed ten randomly assembled groups of 54 genes to determine if their expression pattern might also reflect group differences. We saw no discernible pattern in the dendrograms generated from the random sets of genes and none of the groups were appropriately clustered (data not shown). Thus, the sensitivity of the previously derived GET to reflect tissue specificity and pathological conditions appears to also discriminate anatomically distinct regions within the heart.

### Spatial pattern

We compared the expression of the GET between adult RV and adult LV. There were just 18 genes with significant changes in expression between the two tissues (Additional file 2: Table S2). We used Ingenuity Pathway Analysis (IPA, Ingenuity Systems, Redwood CA, USA) to assess the biological roles of these 18 genes. IPA is a bioinformatic software that identifies functional and network relationships that are significantly overrepresented within gene lists. The most significant network function represented by these 18 genes was organ morphology, suggesting that the subset of genes from the GET with changed expression between the right and left ventricles was involved in developing and maintaining structural integrity within the heart.

### Temporal pattern

We examined the temporal development of the right ventricle using fetal tissue (approximately 90 days of gestation), normally developing infant right ventricular tissue, and adult right ventricular tissue. The clustering algorithm accurately placed the right ventricular samples into their appropriate groups (Figure 1). The fetal RV and infant RV samples were more closely associated with each other than to any other samples, suggesting the GET was strongly influenced by maturity of the tissue. In looking at individually significant changes in gene expression, there were 11 genes with altered expression between the fetal RV and infant RV. There were 25 genes that differed in expression between the infant and adult RV, suggesting the homoeostatic patterns reflected by the GET differed much more between the adult and infant RV as compared to the infant and fetal RV. This is reflected in the PCA analysis shown in Figure 2B. The primary component of separation appeared to be related to age, mature vs. young. In addition, the groups had no members that overlapped within two standard deviations, demonstrating the clear discrimination by the GET among groups.

Taken together, there were 32 genes that differed in expression in at least one of the comparisons among the three different normally developing RV datasets (Additional file 2: Table S2). Ontological analysis (using IPA) of these 32 genes revealed the highest associations with the network functions of cell movement, cell death and survival, and molecular transport. The top canonical pathways associations were inhibition of angiogenesis and regulation of actin-based motility. Since these genes represent the stoichiometric changes in the GET during the fetal to adult maturation of the right ventricle, it is perhaps not surprising that the regulation of cell movement and cell survival are predominant categories represented by these 32 genes.

### Pathological pattern

The patterns from the right ventricular tissue from infants with TOF all grouped together with the exception of one sample that clustered most closely with the group of control infant RV (discussed above). Interestingly, the TOF RV tissues were more closely associated with adult LV and RV than to the age-matched controls. The TOF tissue was distinctly different from similarly aged, normally developing RV with 33 of the 54 genes examined having a statistically significant change in expression (Additional file 2: Table S2). This is perhaps not surprising for a tissue with developmental disarray resulting in dramatically different physiological conditions compared to structurally normal tissue.

The most closely clustered group to the TOF patients was the normal adult LV. The right ventricle of infants with TOF is typically hypertrophic. Our gene expression clustering suggests the pathophysiological conditions experienced by the RV resulting from TOF may influence gene expression stoichiometry in a way that similarly mimics the physiological conditions of the adult ventricle.

**Figure 3:**
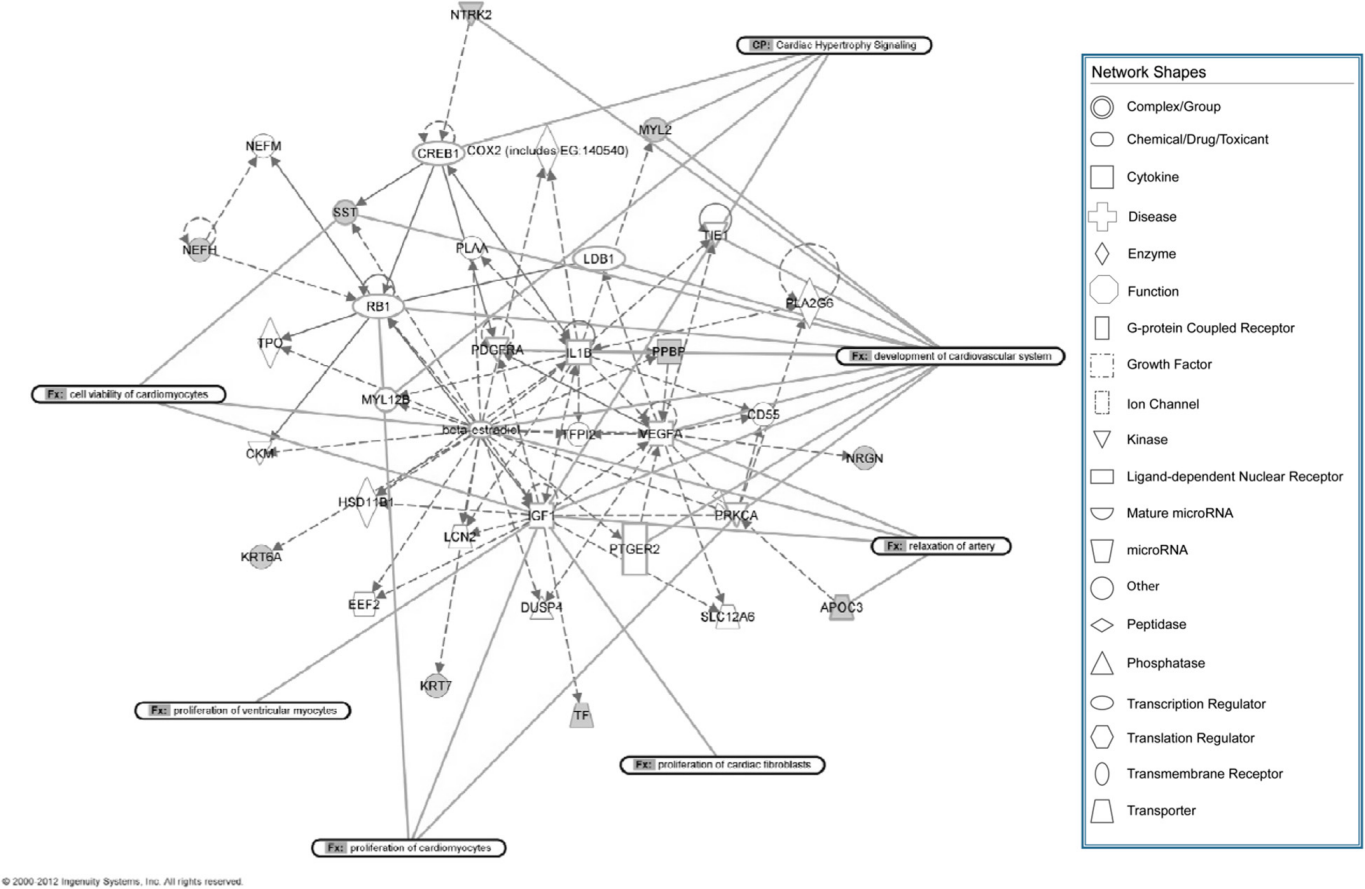
**Interactive network of GET genes with shared expression patterns between TOF RV and adult LV.** Interactive network of genes associated with the GET with shared expression patterns in TOF and adult LV compared to normally developing infant RV. Genes in *gray* are from the GET.

There were 19 GET genes with statistically significant changes in expression between TOF RV and normally developing infant RV but were unchanged between TOF RV and adult LV (marked with an ‘x’ in Additional file 2: Table S2). We reasoned that these genes might represent networks functioning in common between TOF and adult LV which might suggest a physiological explanation for the clustering pattern. The most statistically significant functional network identified using IPA (see ‘Methods’ for explanation of bioinformatic assessment) contained 10 of the 19 GET genes and formed an interactive network involved broadly in gene expression, cell signaling, and cell growth. More specifically, several members of this network are known to be involved in cardiac development, myogenesis, and cardiac hypertrophy (Figure 3). These comparisons specifically substantiate the hypothesis that pathological conditions are associated with the significant deviation from normal biochemical stoichiometry which can be represented by the expression patterns of a small number of key genes.

As mentioned above, the 54 genes we used for hierarchical clustering placed one TOF sample with the control infant RV group and the rest of the subjects with TOF clustered as a single group. With respect to the GET correctly predicting the pathological condition, this represents 94% sensitivity (correct grouping of the defect).

Based on our analysis, the GET can be applied to pathologic tissues as well as normal tissues with great accuracy. Most interesting is the difference between the TOF RV and control-matched infant RV. This comparison has the most genes in the GET with a significant change in expression (33 genes, see Additional file 2: Table S2) and directly reflects how the GET can model pathologic tissues. This undoubtedly reflects the consequences of the failure of correct cardiac development in infants with TOF. Furthermore, it may signal the presence of genetic variants that collectively destabilized developmental networks leading to the heart defect. This warrants additional research as it may provide insight for the current difficulty in identifying the genetic basis for most CHDs.

## Conclusions

Our results suggest that even at the intra-organ level, cells differentiate and maintain a biochemical stoichiometry that is reflected in the expression of a small representative sample of genes which likely represents functional networks required for homeostasis. Perhaps not surprisingly, in the pathophysiological conditions resulting from the failure of proper development, homeostatic imbalance is reflected in the disruption of the stoichiometry of the GET. The GET reflects key genetic pathways, whose disturbance may have contributed to the heart defect and/or its physiological consequences. The perturbations in the GET suggest a possible genetic signature that might aid in prognosis and genetic counseling and, with additional study, might contribute to improved therapy.

## Methods

The Children's Mercy Hospitals and Clinics' institutional review board approved this research protocol. The parents of all subjects provided written consent for participation after reading the consent document and having their questions answered.

Our subjects were children less than 1 year of age with TOF requiring surgical reconstruction. The diagnosis and anatomy were obtained by echocardiography and angiography and confirmed at the time of surgery. All microarray analyses were run on samples from sixteen infants with TOF who had normal chromosomes (i.e., without 22q11.2 deletions, as determined by comparative genomic hybridization and/or *in situ* hybridization by the clinical cytogenetics lab at Children's Mercy Hospital), eight infants with normally developing hearts, and three fetal samples (Additional file 1: Table S1). The comparison tissues from the eight normally developing infants (three males, five females) were obtained from LifeNet Health (http://www.lifenethealth.org; Virginia Beach, VA, USA). LifeNet Health is a non-profit regenerative medicine company that provides bioimplants and organs for transplantation. The comparison samples were matched for age to the study population, and all control subjects expired due to non-cardiac related causes as described in detail previously [13]. We obtained three fetal hearts (approximately 90 days of gestation) through the National Institute of Child Health and Human Development-supported tissue retrieval program from the Central Laboratory for Human Embryology at the University of Washington (Seattle, WA, USA). The fetal hearts were dissected by one of the surgeons who also performed many of the reconstructions of the conotruncal defects (JEO) to ensure that the tissue analyzed was from a similar location as the tissues removed during surgery (as described previously [13]).

In addition, exon array data was downloaded from the GEO derived from the right ventricular tissue of 4 adult subjects [14] and the left ventricular tissue of 14 adult subjects [15]. As an outgroup comparison, exon array data was downloaded from nine noncancerous colon samples [16]. Sex and age of the subjects and GEO accession numbers for all arrays are presented in Additional file 1: Table S1.

### Microarrays

We generated exon array data for this study using RNA extracted from the right ventricular tissue from 16 infants with tetralogy of Fallot, right ventricle from 8 typically developing age- and sex-matched comparison infants, and right ventricle from three fetal hearts (gestational age 90 days as described above). The exon arrays were all Affymetrix HuEx-1_0-st-v2 and gene expression levels were generated as a mean of all probes within the coding sequence of the gene. The raw data from our arrays have been deposited in the GEO (accession number to be supplied upon acceptance). Our arrays were run at the University of Kansas Medical Center-Microarray Facility (KUMC-MF) according to the manufacturer's protocols. The KUMC-MF is supported by the Kansas University-School of Medicine, KUMC Biotechnology Support Facility, the Smith Intellectual and Developmental Disabilities Research Center (HD02528), and the Kansas IDeA Network of Biomedical Research Excellence (RR016475). All of our array data has been deposited in the GEO and will be available after July 2014. However, we make the data available prior to this date upon request.

### Statistical analysis

All statistical analyses of expression patterns were performed using the statistical software: Partek Genomics Suite software version 6.6. Study arrays were uploaded to Partek, and quality assessment and RMA background correction and quantile normalization were applied to ensure unbiased downstream analysis. Unsupervised hierarchical clustering was utilized within the Partek software to group subjects with similar expression patterns based on the set of genes from the gene expression template described by Hwang et al. [12]. No *a priori* grouping was applied. We tested several algorithms for accuracy in clustering: average linkage with squared euclidian distance (averaged point-to-point distances to define cluster to cluster distance), complete linkage without squared euclidian distance (furthest neighbor clustering algorithm uses the furthest distance between points to derive the groups), and single linkage (point-to-point distance of closest neighbors). The complete clustering algorithm without squaring the euclidian distance produced the best dendrogram, accurately reflecting group relationships with only one TOF sample associated with the normal infant RV. The average linkage algorithm correctly clustered most of the groups, but split the LV samples into two subgroups, one group with ten samples that were most closely associated with the TOF samples and four samples that were most closely associated with the RV samples. Adding colon samples also had the effect of causing separation of the LV samples regardless of the clustering algorithm (Additional file 3: Figure S1). This obviously reflects subtle underlying structure within the data. The single linkage clustering algorithm produced no clear pattern among the samples.

Differences in expression were determined using *t* test with significance set at *p* ≤ 0.05 after using the Benjamini-Hochberg correction for multiple testing based on the entire set of genes in an array, as we have done previously [13,17]. Likewise, principal component analysis was performed within Partek using the set of 54 genes for partitioning.

Network and pathways analysis was done using the bioinformatic software IPA which allows statistical assessment of functional associations within gene lists derived from comparative expression analysis.

## Competing interests

The authors declare that they have no competing interests.

## Authors’ contributions

AR participated in the study design, analysis of data and drafting of the manuscript. NK participated in the study design, microarray analysis, generation of GET data, and review of the manuscript. AB participated in analysis of data and review of the manuscript. JM participated in study design, sample collection and review of the manuscript. JEO participated in study design, sample collection and review of the manuscript. DCB participated in the study design, generation and analysis of data and drafting of the manuscript. All authors read and approved the final manuscript.

## Supplementary Material

Additional file 1: Table S1 Sample demographic data. Click here for file

Additional file 2: Table S2 Significant changes in expression among heart tissues (fold change). Click here for file

Additional file 3: Figure S1 Hierarchical clustering based on 54 genes of the gene expression template (GET). The clustering algorithm used a complete linkage algorithm without squaring the euclidian distance to establish the dendrogram. Click here for file
