# Supplementary material for: A tissue-specific gene expression template portrays heart development and pathology
Source: Hum Genomics. 2014 Mar 11;8(1):6. doi: 10.1186/1479-7364-8-6 (PMC4007492; doi:10.1186/1479-7364-8-6)
Supplement: Additional file 1: Table S1 — Sample demographic data. [file 1479-7364-8-6-S1.pdf]

Additional Table 1. Sample Demographic Data*

GEO # / citation	Lab ID #No*.	Sex	Age	Group
GSM414643 / [1]		F	48y	LV normal Adult
GSM414644 / [1]		F	51y	LV normal Adult
GSM414645 / [1]		M	37y	LV normal Adult
GSM414647 / [1]		M	41y	LV normal Adult
GSM414648 / [1]		M	44y	LV normal Adult
GSM414649 / [1]		M	44y	LV normal Adult
GSM414651 / [1]		M	51y	LV normal Adult
GSM414652 / [1]		M	52y	LV normal Adult
GSM414654 / [1]		M	56y	LV normal Adult
GSM414655 / [1]		M	61y	LV normal Adult
GSM805384 / [2]		NA	NA	RV Normal Adult
GSM805387 / [2]		NA	NA	RV Normal Adult
GSM805390 / [2]		NA	NA	RV Normal Adult
GSM805392 / [2]		NA	NA	RV Normal Adult
GSM874677 / [3]	GE001	F	1m	RV Normal infant
GSM874678 / [3]	GE002	M	1y	RV Normal infant
GSM874679 / [3]	GE003	M	2.5m	RV Normal infant
GSM874656 / [3]	GE004	M	1m	RV Normal infant
GSM874657 / [3]	GE005	M	4m	RV Normal infant
GSM874658 / [3]	GE007	F	2m	RV Normal infant
GSM874659 / [3]	GE008	F	2m	RV Normal infant
GSM874660 / [3]	GE009	F	12m	RV Normal infant
GSM874676 / [3]	CR003	F	10m	RV TOF Infant
GSM874675 / [3]	CR013	F	< 12m	RV TOF Infant
GSM874674 / [3]	CR018	M	< 12m	RV TOF Infant
GSM874673 / [3]	CR023	F	< 12m	RV TOF Infant
GSM874664 / [3]	LabCR010	F	7m	RV TOF Infant
GSM874666 / [3]	LabCR012	M	7m	RV TOF Infant
GSM874661 / [3]	LabCR016	F	8m	RV TOF Infant

GSM874665 / [3]	LabCR020	M	7m	RV TOF Infant
GSM874663 / [3]	LabCR022	M	7m	RV TOF Infant
GSM874667 / [3]	LabCR024	M	3m	RV TOF Infant
GSM874668 / [3]	LabCR025	F	4m	RV TOF Infant
GSM874662 / [3]	LabCR039	M	9m	RV TOF Infant
GSM874671 / [3]	LabCR042	M	10m	RV TOF Infant
GSM874672 / [3]	LabCR047	M	1.6y	RV TOF Infant
GSM874669 / [3]	LabCR055	F	8m	RV TOF Infant
GSM874670 / [3]	LabCR057	F	9m	RV TOF Infant
GSM874680 / [3]	FH01	F	94 days gestation	RV Fetal
GSM874681 / [3]	FH46	F	94 days gestation	RV Fetal
GSM874682 / [3]	FH68	M	96 days gestation	RV Fetal
GSM475126 / [4]		F	55y	sigmoid colon
GSM475128 / [4]		M	58y	sigmoid colon
GSM475129 / [4]		M	67y	sigmoid colon
GSM475130 / [4]		M	58y	sigmoid colon
GSM475132 / [4]		M	62y	sigmoid colon
GSM475133 / [4]		F	80y	sigmoid colon
GSM475134 / [4]		F	47y	sigmoid colon
GSM475135 / [4]		F	65y	sigmoid colon
GSM475136 / [4]		F	62y	sigmoid colon

NA = Not Available

*samples with a lab ID # were analyzed in the Bittel lab. Those with only a GEO number and no lab ID # were downloaded from the GEO.

1. Kong SW, Hu YW, Ho JW, Ikeda S, Polster S, et al.: **Heart failure-associated changes in RNA splicing of sarcomere genes**. Circ Cardiovasc Genet 2010, **3**:138-146.
2. Gupta S, Halushka MK, Hilton GM, Arking DE: **Postmortem cardiac tissue maintains gene expression profile even after late harvesting**. BMC Genomics 2012, **13**:26.
3. O'Brien JE, Jr., Kibiryeva N, Zhou XG, Marshall JA, Lofland GK, et al.: **Noncoding RNA expression in myocardium from infants with tetralogy of Fallot**. Circ Cardiovasc Genet 2012, **5**:279-286.

4. Mojica W, Hawthorn L: **Normal colon epithelium: a dataset for the analysis of gene expression and alternative splicing events in colon disease**. BMC Genomics 2010, **11**:5.
