# Supplementary material for: A tissue-specific gene expression template portrays heart development and pathology
Source: Hum Genomics. 2014 Mar 11;8(1):6. doi: 10.1186/1479-7364-8-6 (PMC4007492; doi:10.1186/1479-7364-8-6)
Supplement: Additional file 3: Figure S1 — Hierarchical clustering based on 54 genes of the gene expression template (GET). The clustering algorithm used a complete linkage algorithm without squaring the euclidian distance to establish the dendrogram. [file 1479-7364-8-6-S3.pdf]

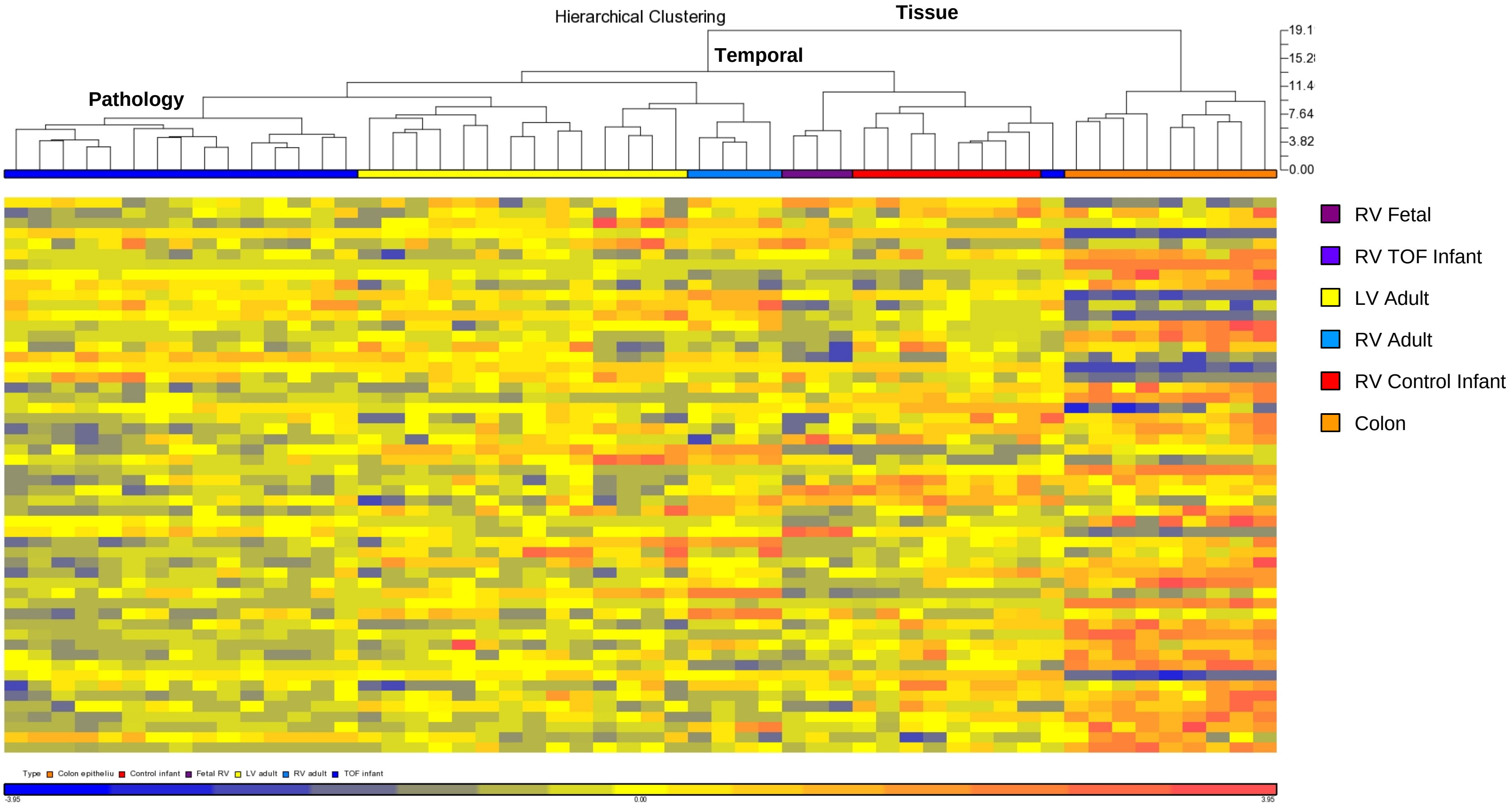

Additional Figure 1. Hierarchical clustering based on 54 genes of the Gene Expression Template (GET). The clustering algorithm used a complete linkage algorithm without squaring the euclidian distance to establish the dendrogram.
